# On Diet, with Its Influence on Man

**Published:** 1845-01

**Authors:** 


On Diet, with its Influence on Man. Being an Address to Parents, &c., or how to obtain Health, Strength, Sweetness, Beauty, Development of Intellect, and Long Life. By *Thomas Parry.* Octavo, pp. 119. Highley. London, Nov. 1844.

To works emanating from regular practitioners, we have seldom seen a more *taking* title prefixed, than the one at the head of this article. The words "sweetness," and "beauty" will attract the attention of a considerable number of damsels and dandies, whose mirrors have sometimes raised suspicions in their minds respecting the sweetness and beauty of their precious persons. We are among the last to under-estimate the influence of diet on mind as well as matter—on health as well as on disease; but we cannot help suspecting that Mr. Parry has overdone his work in the following proposition;—"*take away from your nosology, fevers, miasmatic and contagious—surgical accidents, and specific diseases, and may not nearly all the rest be traced to dietetic errors?*" Mr. Parry has left out the most efficient morbific causes of all—perturbations and anxieties of mind—nakedness and exposure to wet and cold—penury and destitution, &c.! The author of this little work has evidently many peculiar notions about diet, some of which are not very clear or tenable; but many of them are deserving of consideration. The book is divided into thirteen Chapters—some of which we shall notice.

I. *Birth to Weaning.*—Human milk is the proper nourishment for this period, and next to that, the milk of cloven-footed, and cud-chewing animals—especially the cow, whose milk ought to be diluted for young infants, and very little sugar added. The author has never seen any advantage from asses' milk, whether in health or disease. The milk of the ewe is very light and nutritious. "Corn and its products, flour and meal, is universally the just food of human offspring when making its separation from the mother." This is the same in all countries, and has been the same in all ages. Of the varieties of corn, wheat is the most nutritive—containing the greatest quantity of gluten and starch. The abundance of gluten in wheat renders that corn the most nutritive, but most difficult of digestion. The leavening of bread renders it more digestible.

"This leavening, however, is but one of the processes employed for rendering more digestible this dense nutritious food. It is afterwards baked, by which much more of the gluten is destroyed, it being rendered friable, and thus deprived of its gluey nature; and frequently this process is done twice to render the food still more light and easy of digestion. Bread thus prepared was formerly called *bis-cuit*, or twice baked. The term biscuit is now quite misapplied in general usage; the article to which the term is at present appropriated being an unleavened bread only once baked,—a good and nutritious food, but not a light and easy digesting bread, as biscuit is." 9.

Wheat then being more difficult of digestion than other species of corn, barley is preferable for the young stomach, being the most mucilaginous.

"In the first months of infancy, if feeding be at all necessary, and corn be used, it should be either the light flowery oat, or the diffusing starch (arrow-root), or the mucilaginous rice. But if wheat food be preferred from prejudice or custom, then it should be leavened and biscuited, or twice baked—such are rusks, and tops and bottoms; these should never be burnt, although well baked, burning not only making them bitter and offensive to the stomach, but destroying also the nutritive quality of the corn. As the infant advances in age and strength, the degree of leavening and baking may be lessened; and, after a few months, baked flour, flour tied up and boiled in a cloth for several hours, biscuit powder, and leavened bread, may come to be used without so great a destruction of the gluten as had been before necessary." 10.

II. *Nine to Eighteen Months.*—The child being weaned, a more copious supply of the light nutriment alluded to may be allowed. The milk may be less diluted—eggs may be sparingly used in pudding—weak broths may be allowed—but animal food is, as yet, improper. " But if a head-strong, self-willed, and bigoted person must indulge a fancied equality with some other child, who has been imprudently fed, then, in pity to the infant, let the meat be very small in quantity, very much cooked (not burnt) and cut into pieces not larger than a common currant." By these means, thinks our author, " the child will have a good chance of passing it off undigested,"—and " without other ill effect than having lost that nutriment which ought to have been given in its stead." In fine, he thinks that children from nine till eighteen months, should be fed on milk and farinacæ, with some eggs sparingly used. Light broth under particular circumstances. There is much good sense and wise counsel in these observations.

III. *Eighteen Months to Seven Years.*—Towards the close of the eighteen months, our author begins to relax in his farinalactic regimen, and indulges the young omnivorous animal in his carnivorous propensities. He is then running about. The mild nutriment is still to be continued, but " a small quantity of meat well cooked and cut small, may be commenced when the child has cut most of his teeth." This amendment, or rather adaptation of the diet may be gradually increased as the child advances in years. Neither wine, beer, cider, or any fermented drink should be allowed. Under such regimen the child will be lively and inclined to exercise. Ripe fruit may be sparingly admitted to the table of the children at this period.

IV. *Seven to Fourteen Years.*—The growth of the body and the necessary exercises during this septenniad, require a " very plentiful supply of nutriment." The gluten of the wheat is now useful, and heavy substantial pudding may be taken with advantage, especially if the boy's digestion be strong. If the plain pudding lies heavy on the stomach—

" To obviate this evil, and yet avail ourselves of the sustaining gluten of the corn, strongly nourishing and digestible pudding may be made by mixing with the wheat flour, eggs, butter, fat, and milk, to which may be added sugar as a condiment." 20.

The use of fruit must still be sparing ; and cheese, where it agrees, is very nutritious, but difficult of digestion. Beer and wine are unnecessary, except medically, and when taken should be diluted, so as not to produce exhilaration.

V. *Fourteen to Man and Woman-hood.*—In this Septenniad, the growth of the body is still advancing—exercise is considerable, and the expenditure of education is to be supported. An abundant supply of good nutritious food is now indispensible. Discretion in butcher's meat and wine is necessary, upon the following grounds :—

" First, Too low a diet gives an apathy of character, with slowness of action, and weakness of body.

" Secondly, Too high a diet gives impetuosity of passions and temper, oftentimes with cruelty of disposition.

"Thirdly, A diet without exhilaration gives a gloomy tendency, with despondency, instead of buoyancy of spirit." 27.

This being the chief period in which the germs of consumption develop themselves, our author discusses the diet which accelerates or checks that fatal development. The Christian differs from the Hebrew world, and all those who observe the injunctions of the Old Testament, by eating "meat with the blood." The Jews, Turks, Arabians, he says, who avoid blood and swine's flesh, "are infinitely more free from disease than the Christians —more especially do they escape those opprobria of the medical art, *scrofula*, gout, consumption, and madness." We doubt this statement; but, granting that it is perfectly correct, we do not draw the same inference that Mr. Parry does—namely, that such immunity is owing to abstinence from hog's flesh. We believe that Hippocrates was a better physician than Moses; and everybody knows that the Coan Sage spoke highly in favour of the flesh of the porker.

"The swine-fed navies of Christendom suffered greater devastations from a painful tubercular disease of the bowels, (dysentery,) than from any other cause." 28.

The above passage induces us to think that Mr. Parry is not a medical man. The scorbutic dysenteries of seamen would have been cured, instead of being caused by pork, had it been fresh pork; but it was the *salt* beef and *salt* pork that induced scurvy and bowel-complaints. The author has a horror of black-pudding, and of fowls whose necks are wrung, and the blood left in them; but the swine's flesh is abomination! We think he must be an Israelite!

"If this important point be neglected in dieting for sweetness, strength, and beauty, and unclean food be substituted for that which is clean, then, 'it will come to pass,' as formerly said by Isaiah when the Israelites forsook their principles, 'instead of a sweet smell there will be a stink; instead of well-set hair, baldness; and burning (inflammation) instead of beauty.'" 29.

We consider this opinion as a mere prejudice or superstition, without any foundation whatever. We agree with our author, however, in his next proposition, that all violent gymnastic exercises are to be avoided during the third Septenniad. The body is not strong enough, in fibre or texture, to admit of these experiments, and great mischief is annually done in the attempt. If the top of the strength be reached, it can never be held on in any living being, but is always followed by exhaustion and withering of the powers.

VI. *Exhilaration.*—This has the sanction of Scripture, the authority of profane history, and the consent of Reason. "Neither health, society, nor individual happiness, can go on without it." This dogma we much doubt, as far as wine is concerned in exhilaration. This term of our author, however, excludes intoxication—the use, and not the abuse, of wine, being the subject of Mr. Parry's eulogium. Mr. P. has a great aversion to bitters.

"And we now come to an exposition, why bitters came to be used in diet, when the Dutch had sent to us beasts' food to eat, and the natural consequence was experienced in stomach-pains, cholics, and all the catalogue of gastric miseries which men relieved as well as they could by hot spices, spirits, æther, fæti d gums, &c. The stomach of persons thus pained by improper food, and then eased by hot spices, then pained and eased again, soon continually became irritable or easily pained. Now, all bitter extractive, with very little or no exception, is deadening upon animal fibre; it is not narcotic, or sleep-giving, but directly deadening; it kills flies instantly, and shows its mortal powers very quickly upon most small animals; when much used in medicine, it too often proves fatal,—as was the case some years ago with the Portland powder (a strong bitter), and as has been the case too often lately in the use of gentianine, and other very concentrated bitters. These things prove at first very deadening to stomach irritation and pain, relieve the patient from immediate suffering, but ultimately, if persisted in, deaden him altogether; although from the absence of premonitory symptoms, the treacherous bitter is rarely suspected. The introduction of bitter into diet was, then, from the same people from whom we received the garden-food, so calculated to pain, irritate, and injure the stomach, experience having taught them its use upon their own badly nourished bodies—this bitter was the hop. It soon followed garden-stuff; and bitter extractive in some form, has ever since been a portion of the drink, and a principal ingredient in doctors' stuff." 52.

The doctors come in for a share of Mr. Parry's ire against bitters.

"These hot condiments and bitter drugs and liquid fire, were not all that was needed; bowel-purges of all kinds came to be much wanted; and in the severe pains, frightful spasms, convulsions, and horrible distortions of anguish, which characterize the diseases now induced, those which purged most rapidly were most appreciated, whether from the poisonous mineral, the scouring wild plants, the griping gums, or the tormenting resins. And the great sum of stomachic physicking lay, and has since laid, in clearing the bowels, giving hot spices or aromatics and drugging with bitter." 54.

The "cursed weed" meets with mercy in the hands of our author. "By this herb, the hungry cravings of an ill-nourished body are assuaged —the irritated nerves of an overworked man are soothed—and the wretched victim of a bad government indulges in dreams he cannot rationally hope."

Mr. Parry tells us that, before *mirth* can be brought out, *cheer* must be put in. The body should be well nourished, before the exhilarating materials are imbibed, otherwise even wine may fail to produce merriment.

"The degree of nourishing required, preparatory to exhilaration, is greatly beyond what the medical world has of late years deemed prudent. The system should have some degree of *embonpoint;* there should be a degree of fatness about the eyes, by which, when the person laughs, wrinkles are made on the outer sides of the orbit; the merry and cheerful man is thus easily discerned: and this is a leading feature known to characterists, or they who depict characters, whether painters or actors. A leanness about the eye, from which it sinks, and a flattened skin upon the prominent outer part of the orbit, is the sign of inanition, weakness, distress, and a leading outline of death, and also a never-failing mark of him who drinks instead of eats. 'Laugh and grow fat,' is a common phrase in England; but the order of events is the reverse, and the phrase should be 'Grow fat and laugh.'" 58.

Our author advises every man and woman to exhilarate with good wine, the genuine product of the vine, if possible—if not, with fictitious wine or fermented liquors from sugar-fruits, corn, or even sugar-roots—carefully avoiding all bitters. Next to wine, brandy, diluted largely with water, is the best drink. The catalogue of diseases from intemperance is frightful, but scarcely exaggerated.

"Should the badly managed man escape these palpable manifestations of disease, there is one suffering or blot in his being he cannot miss. He must inevitably become enervous, or enervated; that is, defective in courage, hesitating in decision, irresolute in action." 59.

Well, the farrago of anti-nervous and stinking medicines may be thrown to the dogs, or rather to the cats. "The only remedy is wheaten bread." "The only permanent tonic to the nervous system is corn." When, says our author, the divine, physician, singer, player, become nervous in their public exhibitions, it is because they doubt their powers and hesitate. "They who are well corned, like the highly-fed horse, doubt not." Finally, says he, "the great bulk of enervous cases are dependent upon insufficient corn." 61. Here is a new argument against the present corn-laws, and an excellent topic for declamation by the LEAGUE! We thus see that modern science has verified the old law, that "bread is the staff of life."

Our author disapproves of the old adage—"eat when hungry, drink when dry." Regular meals, he thinks, should be taken at regular periods, paying no attention to appetite. He is right. Much has been said, and uselessly said, respecting the quantity of our food. No two people require the same amount. Leave off before satiety, and eat slow. That sentence comprises the golden rule. The *quality* may be daily varied—but the *quantity* never exceeded. Rest after the meal of animal food seems necessary in all weakly persons. Digestion is much impeded by exercise.

"The combination of a late dinner and a heavy meat supper is fraught with apoplexy and many horrible effects, as nightmare, frightful dreams, &c. Further, the active man will find it imperative upon him to be systematic in all the parts of his dieting, and he will do well not to submit to temptations which may present themselves. Bread, as often as he pleases in the morning, meat once in the even, exhilaration occasionally at night, is the system he must act upon. If the method be reversed, and exhilaration be made in the morning, he will frustrate all." 68.

The following passage is amusing, and there is much truth mixed with error in it.

"I shall conclude this portion of my writing by waiving all anatomical descriptions, all physiological discoveries, and all medical theories and hypotheses. The grounds excluding these are, that all stomachic theorists become dyspeptic; and the body corporate of physic, who have most of this knowledge, are the most dyspeptic portion of the people; that since Dr. Fordyce's clever book upon digestion, indigestion has increased wonderfully, and is still increasing. Anatomy, dating from Vesalius, has had little or no connection either with diet or physic in practice. The College of Physicians and Company of Apothecaries know this; inasmuch as they compel all students to study physic from Greek and Latin authors, who knew nothing either of anatomy or physiology. Experience, observation, and natural good sense, were sufficient to produce the greatest practical men we are acquainted with; and although, as a surgeon, I highly value anatomy, and, as a philosopher, I have an intense interest in physiology, chemistry, and other branches of the profession, yet, as a dietician, I know them to be worse than useless: for the slightest error in induction upon the facts of those sciences leads to errors fatal to strength, health, and life; and I have arrived at the conviction that a knowledge of the qualities of various articles of diet is more important than a knowledge of structure and function, because it alone involves the practice of the dietician." 69.

VII. *Labourers' Diet.*—To meet his hard, long, and fatiguing work, the labourer should make his prop or support the highest coarse corn of the country in which he lives—always preferring wheat. If it be what is termed heavy, close, compact, it may be a little more indigestible ; but it will be nutritious. Still he imperatively demands and requires "*flesh,* for the temper of his body ;" and, unless he obtains a fair proportion of this, he becomes a premature infirm old man. Swine's flesh, of course, is objected to by our author ; but, under existing circumstances, it is impossible for the poor man often to get anything better than pork and bacon—not always even that! Our author recommends the labourer to eat very little salt with his meat, and, if he can, to return to the old custom of his ancestors —"giving the zest or relish with sugar." For drink, he advises the hard labourer to use water—sugar and water—or "sweet fresh beer." Bitters, of course, are prohibited.

"Neither should the labourer, when thirsty, ever drink hastily; it makes a man not firm in principle or act, to do thus. I have known several deaths from this error, and shall therefore persuade the labourer, when parched with thirst and tempted by a cooling stream, to lap like a dog, not draught it in like a cow." 75.

His toil over, the labourer may, if he can, exhilarate, but not get drunk, on good ale—especially of his own brewing.

"Under irritation or over-excitement, he may cautiously smoke tobacco, and *exhilarate when recovered;* but to smoke and drink together is tomfool's waste, and contrary to sound sense; tobacco being a sedative, and spirit a stimulant, and the combined effect generally intoxication, or poisoning, and sure poverty." 77.

VIII. *Active Professional Man's Diet.*—This man, whose mind and body are strained in the multiplicity of his occupations, will fail in health and powers, if he make not his dependence for sustainment, prop, or support, "upon a plentiful supply of wheat food." "The basis of an active man's diet must be corn." Soups and meat broths, however, are useful and nutritious, when used as condiments to bread. Our author does not approve of strong concentrated soups—weak broths are preferable. But, although he makes bread the staff of life among professional men as well as others, he admits the auxiliaries of meat, fish, game, &c. in moderation. We have only room for one more short extract.

IX. "In dieting for intellectual attainment, great support *should be made upon corn food,* while a very moderate tempering only should be given with meat. Exercise should be regular, but very short of actual fatigue. As great freedom of air should be given as possible, and sleep should be rather prolonged. Luther wrote his great work upon bread and water, Newton his work upon light upon bread and water, and Byron his best productions upon biscuit and water, and, in Spain, the muleteers follow the mules forty and fifty miles a-day upon bread and onions or grapes. It is good food for the intellectual or not over driven man." 118.

We have extended our notice of this little work farther, perhaps, than our readers may deem it necessary; but there is more of eccentricity and novelty in it than usually falls to the lot of much larger tomes that successively take their stand on our library table. Many of Mr. Parry's dietetic opinions are opposed to the generally-received dogmas of the Doctor and the public : but it does not follow that they are therefore baseless or erroneous. The book is well worthy of perusal, and we think some good advice is contained in it.

